# Targeted clearance of *p21*‐ but not *p16*‐positive senescent cells prevents radiation‐induced osteoporosis and increased marrow adiposity

**DOI:** 10.1111/acel.13602

**Published:** 2022-04-01

**Authors:** Abhishek Chandra, Anthony B. Lagnado, Joshua N. Farr, Madison Doolittle, Tamara Tchkonia, James L. Kirkland, Nathan K. LeBrasseur, Paul D. Robbins, Laura J. Niedernhofer, Yuji Ikeno, João F. Passos, David G. Monroe, Robert J. Pignolo, Sundeep Khosla

**Affiliations:** ^1^ 6915 Department of Physiology and Biomedical Engineering Mayo Clinic Rochester Minnesota USA; ^2^ 6915 Robert and Arlene Kogod Center on Aging Mayo Clinic Rochester Minnesota USA; ^3^ 6915 Division of Endocrinology Mayo Clinic Rochester Minnesota USA; ^4^ 6915 Department of Physical Medicine and Rehabilitation Mayo Clinic Rochester Minnesota USA; ^5^ 5635 Institute on the Biology of Aging and Metabolism Department of Biochemistry, Molecular Biology and Biophysics University of Minnesota Minneapolis Minnesota USA; ^6^ Department of Pathology and Laboratory Medicine University of Texas Health Science Center San Antonio Texas USA

**Keywords:** bone, radiation, senescence

## Abstract

Cellular senescence, which is a major cause of tissue dysfunction with aging and multiple other conditions, is known to be triggered by p16^Ink4a^ or p21^Cip1^, but the relative contributions of each pathway toward inducing senescence are unclear. Here, we directly addressed this issue by first developing and validating a *p21*‐*ATTAC* mouse with the *p21^Cip1^* promoter driving a “suicide” transgene encoding an inducible caspase‐8 which, upon induction, selectively kills p21^Cip1^‐expressing senescent cells. Next, we used the *p21*‐*ATTAC* mouse and the established *p16*‐*INK*‐*ATTAC* mouse to directly compare the contributions of p21^Cip1^ versus p16^Ink4a^ in driving cellular senescence in a condition where a tissue phenotype (bone loss and increased marrow adiposity) is clearly driven by cellular senescence—specifically, radiation‐induced osteoporosis. Using RNA in situ hybridization, we confirmed the reduction in radiation‐induced *p21^Cip1^*‐ or *p16^Ink4a^*‐driven transcripts following senescent cell clearance in both models. However, only clearance of p21^Cip1^+, but not p16^Ink4a^+, senescent cells prevented both radiation‐induced osteoporosis and increased marrow adiposity. Reduction in senescent cells with dysfunctional telomeres following clearance of p21^Cip1^+, but not p16^Ink4a^+, senescent cells also reduced several of the radiation‐induced pro‐inflammatory senescence‐associated secretory phenotype factors. Thus, by directly comparing senescent cell clearance using two parallel genetic models, we demonstrate that radiation‐induced osteoporosis is driven predominantly by p21^Cip1^‐ rather than p16^Ink4a^‐mediated cellular senescence. Further, this approach can be used to dissect the contributions of these pathways in other senescence‐associated conditions, including aging across tissues.

## Graphical abstract

1. We generated a new mouse model (*p21‐ATTAC*) for clearance of senescent cells expressing *p21^Cip1^*.
2. Clearance of *p21^Cip1^*‐expressing senescent cells, but not of *p16^Ink4a^*‐expressing cells, prevented bone loss following focal radiation.
3. The genetic approach described here can be used to dissect the contributions of *p21^Cip^*‐ versus *p16^Ink4a^*‐driven cellular senescence in other senescence‐associated conditions, including aging across tissues.

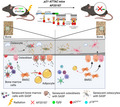

**Abbreviations**
ANOVA: analysis of variance
Ad.N: adipocyte number
Ad.V: adipocyte volume
ATM: ataxia telangiectasia mutated
ATTAC: apoptosis through targeted activation of caspase
BMA: bone marrow area
BV/TV: bone volume fraction
CDK: cyclin‐dependent kinase
Conn.Dens.: connectivity density
DDR: DNA damage response
EGFP: enhanced green fluorescence protein
FRT: focal radiation treatment
MAR: mineral apposition rate
OCY: osteocyte
RNA‐ISH: RNA in situ hybridization
SASP: senescence‐associated secretory phenotype
SMI: structural modal index
TAF: telomere associated foci
Tb.N: trabecular number
Tb.Th.: trabecular thickness
Tb.Sp.: trabecular separation
TIF: telomere dysfunction‐induced foci
vBMD: volumetric bone mineral density
µCT: microcomputed tomography

## 1 INTRODUCTION

Cellular senescence is a non‐proliferative and apoptosis‐resistant state, which can be induced by different stressors including oxidative DNA damage, mitochondrial stress, proteostasis, and other stimuli and is characterized by the production of pro‐inflammatory and matrix‐degrading proteins that are components of the senescence‐associated secretory phenotype (SASP) (Tchkonia et al., 2013). Senescent cells play important roles in tumor suppression, development, and tissue regeneration (Tchkonia et al., 2013). During normal physiological conditions, particularly in youth, senescent cells are cleared by the immune system (Prata et al., 2018). However, with a waning immune system and excessive accumulation of senescent cells with increasing age, disease, and post‐therapy (e.g., radiation or chemotherapy), senescent cells are deleterious and associated with tissue dysfunction and overall morbidity (Khosla et al., 2020).

The induction of cellular senescence following the DNA damage response (DDR) is largely regulated by two pathways, ataxia telangiectasia mutated (ATM)/p53/p21^Cip1^ and p16^INK4a^/Rb (Tchkonia et al., 2013). The DDR stabilizes the tumor suppressor protein, p53, thus activating the cyclin‐dependent kinase (CDK) inhibitor (CDKi), p21^Cip1^, and inducing cell cycle arrest (el‐Deiry et al., 1993; Stein et al., 1999). p16^INK4a^ has been shown to directly bind and inhibit the catalytic activity of CDK4 (Serrano et al., 1993) and CDK6 (Alcorta et al., 1996). Activation of p21^Cip1^ or p16^Ink4a^ and physical binding to D type cyclin and CDK4/6 subsequently activates the tumor suppressor retinoblastoma protein (pRB) (Tchkonia et al., 2013).

Targeted clearance of *p16^Ink4^* ‐expressing cells in *p16*‐*INK*‐*ATTAC* (*p16^Ink4a^* apoptosis through targeted activation of caspase) mice in the presence of AP20187 (a synthetic drug that dimerizes a membrane‐bound myristoylated FK506‐binding protein‐caspase‐8 [FKBP–Casp8] fusion protein) has been successfully used as a strategy to understand the effect of clearing *p16^Ink4a^*‐expressing senescent cells on multiple age‐related conditions, including osteoporosis, frailty, cardiovascular disease, and metabolic dysfunction (for reviews, see (Tchkonia et al., 2013) and (Khosla et al., 2020)). However, although the roles of p21^Cip1^ or p16^Ink4a^ as markers of senescence have been well described (Baker et al., 2011, 2013), their individual contributions in driving senescence more generally are largely unknown.

In the present study, we directly address this issue by first constructing and validating a new mouse model, *p21*‐*ATTAC*. Then, in parallel study designs using the *p21*‐*ATTAC* and the analogous *p16*‐*INK*‐*ATTAC* mouse (Baker et al., 2011), we compare the effects of clearing either *p21^Cip1^*‐ or *p16^Ink4a^*‐expressing senescent cells in a condition where a tissue phenotype (bone loss and increased marrow adiposity) is clearly driven by cellular senescence—specifically, radiation‐induced osteoporosis (Chandra et al., 2020). These studies both dissect the relative roles of the p21^Cip1^ and p16^Ink4a^ pathways in causing radiation‐induced osteoporosis and also establish a system where these mice can be used to evaluate the relative contributions of these pathways in causing senescence in other conditions, including age‐related disorders.

## 2 RESULTS

### 2.1 Generation and validation of the *p21‐ATTAC* model

Details regarding the generation of the *p21*‐*ATTAC* mice are provided in the Methods. Briefly, Figure S1a provides a schematic of the model, where the *p16^Ink4a^* promoter is replaced by a 3.2‐kb genomic fragment containing promoter sequences directly upstream of the mouse *p21^Cip1^* transcriptional start site (López‐Domínguez et al., 2021). This 3.2‐kb fragment contains the appropriate sequences necessary for induction of *p21^Cip1^*, including three p53‐binding sites, which has been validated both *in vitro* (el‐Deiry et al., 1995) and *in vivo* (Wang et al., 2021); further validation of this promoter is also provided below. As in the *p16*‐*INK*‐*ATTAC* model, in the *p21*‐*ATTAC* mice the *p21^Cip1^* promoter drives the FKBP–Caspase‐8 fusion protein, ATTAC, allowing for selective elimination of senescent cells expressing high levels of *p21^Cip1^* following treatment with AP20187, a synthetic drug with no known off‐target effects. Mice expressing this transgene were then generated using site‐specific integration into the *Rosa26* locus (Figure S1b), as described in the Methods.

Prior to further characterization, we evaluated whether treating young (3–6‐month old) *p21*‐*ATTAC* mice with a low senescent cell burden but basal expression of *p21^Cip1^* across tissues with AP20187 would have any adverse effects. For this, we performed a dose‐escalation study (Figure S1c), where the final dose was the 10 mg/kg of AP20187 used in the subsequent experiments. All mice tolerated this dose–response study with no abnormal physical signs and normal weight curves (Figure S1d). In addition, at sacrifice, detailed pathological examination found no abnormal pathologies across tissues in the AP20187‐ versus the vehicle‐treated mice.

**FIGURE 1:**
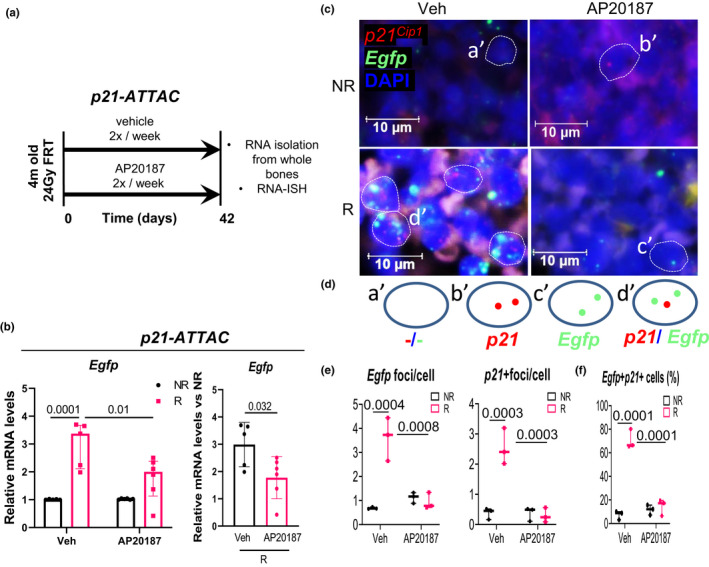
Validation of the *p21‐ATTAC* mouse model using radiation as an inducer of senescence. (a) Schematic showing the experimental design for the *p21‐ATTAC* mice. The right legs of the mice were radiated (24 Gy) near the femoral metaphysis (5 mm above the growth plate), while the left leg served as control. Starting from day 1 post radiation, the animals received vehicle or AP20187 for 2 times per week for 6 weeks. Radiated (R) and nonradiated (NR) femurs at 42 days post‐radiation were collected for qRT‐PCR. (b) The *Egfp* transgene was activated in R bones, and the expression levels were significantly reduced in the AP20187‐treated animals. Statistical comparisons between the groups (left panel in b) was done by an ordinary two‐way ANOVA, with a Tukey’s post‐hoc analysis. (Right panel in b) The *Egfp* expression was normalized to the NR control leg for each animal. Statistical comparison was done using a two‐tailed unpaired *t* test between the Veh‐R and AP20187‐R bones. (c) RNA‐in situ hybridization (RNA‐ISH) was performed using probes against *p21 ^Cip1^* (shown in red) and *Egfp* (shown in green) transcripts. (d) Four populations of bone marrow cells, one with DAPI alone (a′), second expressing *p21 ^Cip1^* (b′), a third expressing *Egfp* (c′), and a fourth expressing both *p21 ^Cip1^* and *Egfp* (d′) were used to generate two kinds of data, one quantifying *Egfp* and *p21 ^Cip1^* foci per cell as shown in (e), and second quantifying percentage of bone marrow cells that expressed *p21 ^Cip1^* and *Egfp*, *Egfp* alone and *p21 ^Cip1^* alone (f). Statistical comparisons were calculated using an ordinary two‐way ANOVA, with a Tukey’s post‐hoc analysis

We next performed both an *in vivo* validation of the promoter used in the *p21*‐*ATTAC* construct and also a side‐by‐side comparison of the induction of the *p21^Cip1^* versus the *p16^Ink4a^* promoters in a model known to induce both genes, radiation‐induced osteoporosis (Chandra et al., 2020). Because the *ATTAC* construct contains an *Egfp* reporter (Figure S1a), the expression of *Egfp* is specific for activation of the transgene in each model. For these and the subsequent studies, 24 Gy focal radiation treatment (FRT) was delivered to a 5 mm area of the right femoral metaphysis and the *p21*‐*ATTAC* and *p16*‐*INK*‐*ATTAC* mice were grouped into those receiving twice weekly doses of either vehicle or AP20187 (Figures 1a and S2a); bones were analyzed on day 42 post‐FRT. In this and the subsequent experiments, the left femoral metaphysis served as the non‐radiated control, as our previous studies have demonstrated that localized FRT has no bone‐damaging effects on the contralateral femurs (Chandra et al., 2014, 2017, 2019). As shown in Figures 1 and S2, radiation treatment resulted in equivalent induction of both the *p21^Cip1^* (Figure 1b) and *p16^Ink4a^* (Figure S2b) promoters. Moreover, treatment of both mouse lines with AP20187 resulted in similar reductions in the *Egfp* transcript, consistent with clearance of high *p21^Cip1^*‐ or *p16^Ink4^*‐expressing senescent cells in the *p21*‐*ATTAC* (Figure 1b–f) and *p16*‐*INK*‐*ATTAC* mice (Figure S2b). Further, RNA in situ hybridization (RNA‐ISH) analysis of bone marrow cells in radiated bones clearly indicated that *Egfp* transgene foci and the endogenous *p21^Cip1^* foci were significantly lower in the AP20187‐treated mice (Figure 1c–e), and majority of the *Egfp* transgene expressing cells also co‐expressed the endogenous *p21^Cip1^* (Figure 1f).

### 2.2 Targeted removal of *p21^Cip1^*‐, but not *p16^Ink4a^*‐expressing cells reduces senescent cell burden and SASP following radiation

To further validate the *p21*‐*ATTAC* mice, we used RNA‐ISH, which demonstrated the presence of three populations of senescent cells (*p21^Cip1^*‐expressing, *p16^Ink4a^*‐expressing, and *p21^Cip1^*/*p16^Ink4a^* dual‐expressing) (Figure S3a). AP20187 treatment of the *p21*‐*ATTAC* mice triggered a 5.4‐fold decline in the *p21^Cip1^*‐expressing bone lining cells (Figure S3b), a 3.4‐fold decline in the *p21^Cip1^*‐expressing bone marrow cells (Figure S3b), and a 41% decline in the *p21^Cip1^*‐expressing osteocytes (Figure S3c,d) as compared to vehicle treatment. There was not a significant reduction in the *p16^Ink4a^*‐ or *p21^Cip1^*/*p16^Ink4a^* dual‐expressing bone lining cells (Figure S3b,d), although the percentage of *p21^Cip1^*/*p16^Ink4a^* dual‐expressing cells was relatively low, limiting the power of detecting changes in this population. Furthermore, AP20187 treatment resulted in reduced *p16^Ink4a^* expression in the radiated bones of the *p16*‐*INK*‐*ATTAC* mice as compared to the vehicle‐treated mice (Figure S4e), with a trend for a decrease in *p16^Ink4a^* + bone lining cells (Figure S4c) but no change in the *p21^Cip1^* + bone lining cells (Figure S4d).

**FIGURE 2:**
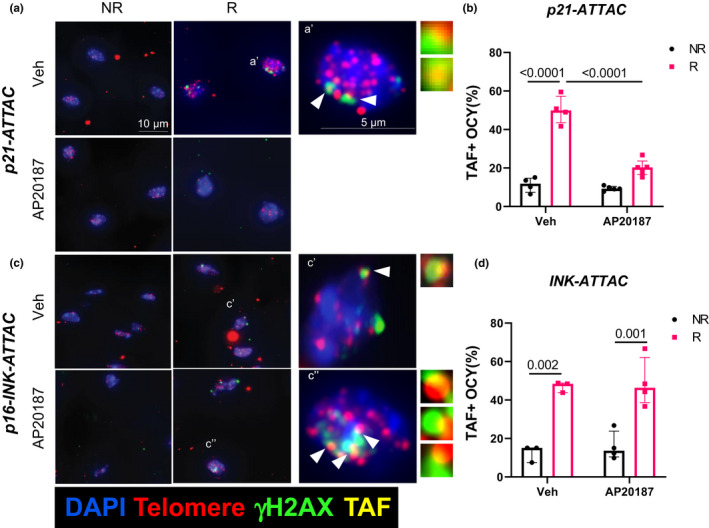
Radiation‐induced senescent osteocytes with dysfunctional telomeres following clearance of *p21 ^Cip1^*‐ and *p16^Ink4a^*‐expressing cells. The right legs of *p21‐ATTAC* mice were radiated (24 Gy, R) near the femoral metaphysis (5 mm above the growth plate), while the left leg (NR) served as control. The animals were assigned to vehicle (*n* = 4) or AP20187 (*n* = 5) treated groups. For the *p16‐INK‐ATTAC* mice, animals received the radiation dose identical to the *p21‐ATTAC* mice and were assigned to vehicle (*n* = 3) or AP20187 (*n* = 4) treated groups. R‐ and NR‐femurs at 42 days post‐radiation were collected and processed for MMA embedding. (a,c) 5 μm deplasticized sections were processed for TAF staining and TAFs were detected. Telomeres (red) and γH2AX (green) are shown in osteocytes (OCY) of non‐decalcified R femurs 42 days post‐radiation. The co‐localization is indicated by yellow TAF foci. Representative images of TAF+ (NR and R) osteocytic nuclei are shown and TAF+ osteocytes were quantified in NR and R femurs of vehicle‐ and AP20187‐treated animals (b,d). Statistical comparisons were calculated using an ordinary two‐way ANOVA, with a Tukey’s post‐hoc analysis

When exposing cells to DNA damaging agents, such as radiation, damage at telomeres is less efficiently repaired compared with the rest of the genome (Fumagalli et al., 2012; Hewitt et al., 2012). Therefore, telomeric lesions are extremely long‐lived and in fact have been shown to persist for months to years both *in vitro* and *in vivo* (Anderson et al., 2019). Telomere‐associated foci (TAF; also known as telomere dysfunction‐induced foci [TIF]), which assess telomeric DNA damage, are increasingly considered the most definitive marker of senescence across tissues, including in bone cells (Chandra et al., 2020; Farr et al., 2016; Wang et al., 2012). We evaluated TAF+ osteocytes as they represent the largest fraction of bone cells and have been implicated as a major senescent cell component in age‐related bone loss (Farr et al., 2017). As expected, the percentage of TAF+ osteocytes increased markedly in the radiated compared with the non‐radiated bones in both the *p21*‐*ATTAC* (Figure 2a,b) and the *p16*‐*INK*‐*ATTAC* mice (Figure 2c,d). However, treatment with AP20187 resulted in clearance of senescent osteocytes, reflected by a significant reduction in TAF+ osteocytes, in the *p21*‐*ATTAC* (Figure 2b) but not the *p16*‐*INK*‐*ATTAC* (Figure 2d) mice. These data thus indicate that, in radiation‐induced osteoporosis, clearance of *p21^Cip1^*‐, but not *p16^Ink4a^*‐expressing cells results in a reduction in senescent osteocytes in bone.

**FIGURE 3:**
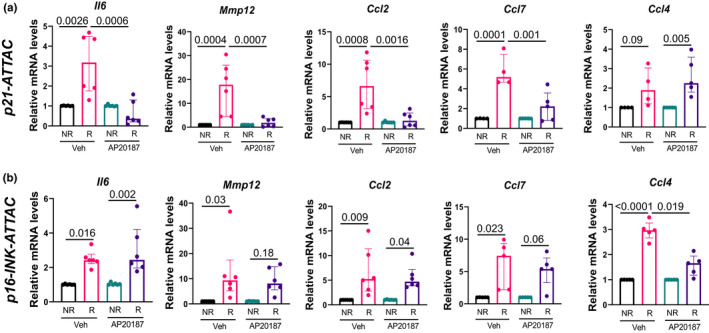
Assessment of radiation‐induced SASP following clearance of *p21^Cip1^*‐ and *p16^Ink4a^*‐expressing cells. The right legs of the *p21‐ATTAC* and *p16‐INKATTAC* mice were radiated (24 Gy) near the femoral metaphysis (5 mm above the growth plate), while the left legs served as control. The animals were assigned to vehicle‐ or AP20187‐treated groups. mRNA from R‐ and NR‐femurs at 42 days postradiation were collected for qRT‐PCR (*n* = 5/group). Gene expression was assessed in NR‐ and R‐bones of both *p21‐ATTAC* (a) and *p16‐INK‐ATTAC* (b) mice, using primers against *Il6*, *Mmp12*, *Ccl2*, *Ccl7*, and *Ccl4*. Statistical comparisons were calculated using an ordinary two‐way ANOVA, with a Tukey’s post‐hoc analysis

Senescent cells are associated with the expression of pro‐inflammatory SASP markers (Coppe et al., 2010). A reduction in SASP factors was one of the key findings associated with the pharmacological clearance of senescent cells by the senolytic cocktail of Dasatinib and Quercetin (D+Q) in age‐ (Farr et al., 2017) and radiation‐related bone loss (Chandra et al., 2020), as well as during genetic clearance of *p16^Ink4a^*‐expressing senescent cells in *p16*‐*INK*‐*ATTAC* aged mice (Farr et al., 2017). Thus, we evaluated a panel of SASP factors that we have previously found to be upregulated following radiation in bone (Table S1) (Chandra et al., 2020) and Figure 3 shows the SASP factors that were downregulated following AP20187 treatment in either the *p21*‐*ATTAC* or the *p16*‐*INK*‐*ATTAC* mice. Thus, *Il6*, *Mmp12*, *Ccl2*, and *Ccl7* were significantly reduced upon AP20187 treatment in radiated bones of *p21*‐*ATTAC* mice (Figure 3a), but not in *p16*‐*INK*‐*ATTAC* mice (Figure 3b). By contrast, *Ccl4* was reduced in *p16*‐*INK*‐*ATTAC* mice following AP20187 treatment in the radiated bones, but not in *p21*‐*ATTAC mice*.

### 2.3 Targeted removal of *p21‐*, but not *p16‐*expressing senescent cells prevents radiation‐induced bone loss

**FIGURE 4:**
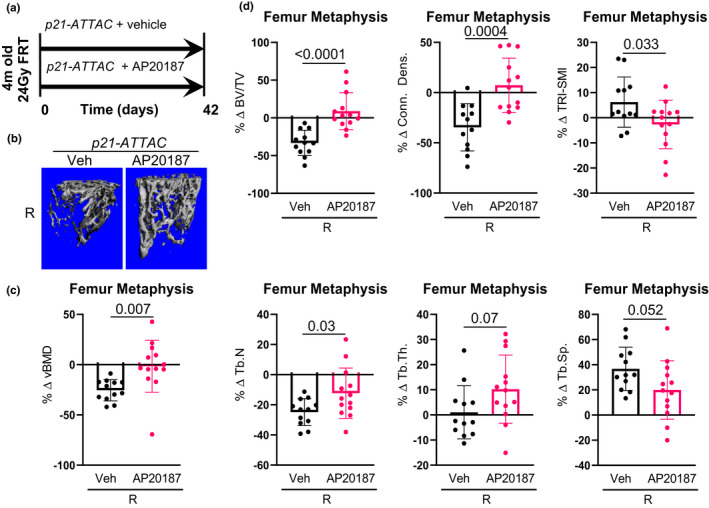
Targeted clearance of *p21^Cip1^*‐expressing cells prevents radiationinduced bone damage. (a) Schematic of the experimental design. The right legs of the *p21‐ATTAC* mice were radiated (24 Gy) near the femoral metaphysis (5 mm above the growth plate), while the left legs served as control. The animals were assigned to vehicle‐ (*n* = 12) or AP20187‐ (*n* = 13) treated groups receiving doses twice weekly for 6 weeks. R‐ and NR‐femurs at 42 days post‐radiation were collected for μCT analysis. (b) Images are 3‐dimensional representations of the bone architecture generated by *ex vivo* μCT scans. All quantifications are done as percentage difference between the R leg vs. the NR control leg and comparisons are between vehicle‐ and AP20187‐treated radiated bones. (c) Percentage change of volumetric bone mineral density, and (d) bone architecture parameters: bone volume fraction (BV/TV), connectivity density (Conn.Dens.), structural modal index (SMI), trabecular number (Tb.N), trabecular thickness (Tb.Th.) and trabecular separation (Tb.Sp.). *p*values were calculated using a two‐tailed unpaired *t* test between the Veh‐R and AP20187‐R bones

**FIGURE 5:**
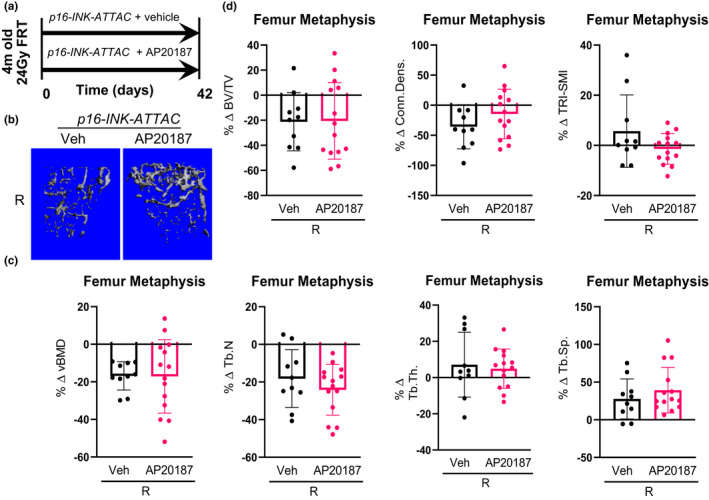
Targeted clearance of *p16^Ink4a^*‐expressing cells has no effect on radiation‐induced bone damage. (a) Schematic of the experimental design. The right legs of *p16‐INK‐ATTAC* mice were radiated (24 Gy) near the femoral metaphysis (5 mm above the growth plate), while the left legs served as control. The animals were assigned to vehicle‐ (*n* = 10) or AP20187‐ (*n* = 14) treated groups receiving doses twice weekly for 6 weeks. R‐ and NR‐femurs at 42 days postradiation were collected for μCT analysis. (b) Images are 3‐dimensional representations of the bone architecture generated by *ex vivo* μCT scans. All quantifications are done as percentage difference between the R leg vs. the NR control leg and comparisons are between vehicle‐ and AP20187‐treated radiated bones. (c) Percentage change of volumetric bone mineral density, and (d) bone architecture parameters: bone volume fraction (BV/TV), connectivity density (Conn.Dens.), structural modal index (SMI), trabecular number (Tb.N), trabecular thickness (Tb.Th.) and trabecular separation (Tb.Sp.). Statistical comparison was done using a two‐tailed unpaired *t* test between the Veh‐R and AP20187‐R bones

We next evaluated the skeletal consequences of clearance of either *p21^Cip1^*‐ or *p16^Ink4a^*‐expressing senescent cells. For these studies, *p21*‐*ATTAC* (Figure 4a) or *p16*‐*INK*‐*ATTAC* (Figure 5a) mice were radiated in the right femur metaphysis at age 4 months, treated with vehicle or AP20187, and sacrificed 42 days later. For simplicity, the skeletal analyses are presented in the radiated bone normalized to the contralateral, non‐radiated bone from the same animal. Thus, in the *p21*‐*ATTAC* mice, AP20187 treatment prevented radiation‐induced reductions in volumetric BMD (vBMD; Figure 4b,c) and deterioration in trabecular microarchitectural parameters (Figure 4d). By contrast, consistent with the lack of effect on TAF+ osteocytes (Figure 2c,d), radiation‐induced reductions in vBMD (Figure 5b,c) or trabecular microarchitectural changes (Figure 5d) were not prevented by AP20187 treatment in the *p16*‐*INK*‐*ATTAC* mice. Interestingly, the beneficial effects of senescent cell clearance in the *p21*‐*ATTAC* mice on trabecular microarchitecture were largely through improvements in trabecular thickness, rather than in trabecular number (Figure 4d).

### 2.4 Targeted removal of *p21‐*, but not *p16^Ink4a^*‐expressing senescent cells maintains BMSC cell fate and bone formation

Radiation‐induced DNA damage induces cellular apoptosis (Chandra et al., 2014) and senescence (Chandra et al., 2020) of osteoblasts and osteocytes. These changes in osteoblast viability or function result in reduced mineral apposition rate (MAR) (Chandra et al., 2014, 2018; Oest et al., 2015). We observed a decline in total osteoblasts in radiated bones of both vehicle‐ and AP‐treated *p21*‐*ATTAC* and *INK*‐*ATTAC* mice, while there was no change in osteoclast numbers in any of the groups (Figure S5a,c). However, when MAR was assessed as a measure of functional osteoblasts, clearance of *p21^Cip1^*‐expressing cells induced a restoration of MAR in radiated bones (Figure S5b), while clearance of *p16* ^Ink4a^‐expressing cells had no effect on the MAR in the radiated bones (Figure S5d).

**FIGURE 6:**
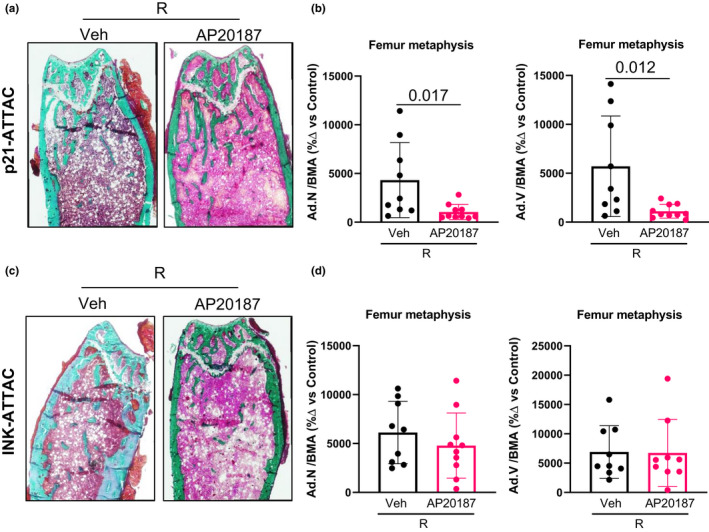
Assessment of marrow adiposity in radiated bones following clearance of *p21^Cip1^*‐ and *p16^Ink4a^*‐expressing cells. The right legs of *p21‐ATTAC* and *p16‐INKATTAC* mice were radiated (24 Gy) near the femoral metaphysis (5 mm above the growth plate), while the left legs served as control. The animals received either vehicle or AP20187 as described above. (a,c) 5 μm Goldner’s trichrome stained MMA‐embedded sections from animals that received either vehicle (*n* = 9) or AP20187 (*n* = 10) in *p21‐ATTAC* mice, and vehicle (*n* = 9) or AP20187 (*n* = 9) in *p16‐INK‐ATTAC* mice, were used to quantify adipocyte numbers (Ad.N) and adipocyte volume (Ad.V) and normalized against bone marrow area (BMA), where BMA is defined as total area minus bone area. Ad.N/BMA and Ad.V/BMA are calculated in vehicle‐ and AP20187‐treated NR‐ and R‐bones. Data from R‐bones are normalized against the control NR‐bones from each animal (b,d). Statistical comparisons were done using a two‐tailed unpaired *t* test between the Veh‐R and AP20187‐R bones

Radiated bones also are reported to have increased bone marrow adiposity (Chandra et al., 2017, 2018; Hui et al., 2012), caused by lineage switching of the bone marrow‐derived mesenchymal stem/stromal cells (BMSCs) from osteoblasts to adipocytes (Chandra et al., 2017), resulting in a significant decline in functional BMSCs and subsequently reducing osteogenic precursors to form new bone. Using bone marrow adiposity as a surrogate for BMSC dysfunction, we found that AP20187 treatment prevented the radiation‐induced increases in bone marrow adiposity (adipocyte numbers and volume) in the *p21*‐*ATTAC* (Figure 6a,b) but not the *p16*‐*INK*‐*ATTAC* (Figure 6c,d) mice.

### 2.5 Further characterization of *p21^Cip1^* + cells following radiation

Given the importance of *p21^Cip1^* + senescent cells in radiation‐induced bone loss, we did additional studies to further characterize these cells. Using RNA‐ISH, we have reported *p21^Cip1^*‐expressing bone lining cells, osteocytes, and bone marrow cells at different time points following radiation (Chandra et al., 2022). Since bone marrow cells are heterogenous in nature, we wanted to identify select *p21^Cip1^*‐ expressing cell types. Using antibodies against cell surface markers and RNA‐ISH for *p21^Cip1^*, we identified elevated levels of *p21*+CD11b+ myeloid cells (Figure S6a,b), *p21*+CD19+ B cells (Figure S6c,d), and *p21*+CD3+ T cells (Figure S6e,f) in radiated bones, on day 7 post‐radiation. Thus, following radiation, multiple cell types (osteocytes, bone lining cells [Figure S3] as well as myeloid cells, B cells, and T cells [Figure S6]) express increased levels of p21.

## 3 DISCUSSION

In the present study, we generated and validated a new mouse model, *p21*‐*ATTAC*, and also used an established mouse model, *p16*‐*INK*‐*ATTAC* (Baker et al., 2011), to directly compare the contributions of p16^Ink4a^‐ versus p21^Cip1^‐driven cellular senescence in a condition where the phenotype is known to be driven, at least in part, by cellular senescence—specifically, radiation‐induced osteoporosis (Chandra et al., 2020). Despite the similar induction of both transgenes in bone by radiation, AP20187 treatment resulted in a reduction in senescent osteocytes and in SASP markers, as well as prevention of both bone loss and increased bone marrow adiposity in the *p21*‐*ATTAC*, but not the *p16*‐*INK*‐*ATTAC*, mice. These findings thus demonstrate that radiation‐induced bone loss is predominantly, if not exclusively, a p21^Cip1^‐driven process. To our knowledge, ours is the first study to use genetic models to dissect p16^Ink4a^‐ versus p21^Cip1^‐driven cellular senescence and provide an approach to further characterize these pathways in other conditions, including the myriad age‐related disorders now associated with cellular senescence (Tchkonia & Kirkland, 2018). From a translational perspective, these studies may also guide the development of novel senolytic drugs that specifically target either p21^Cip1^‐ or p16^Ink4a^‐driven cellular senescence, perhaps allowing for more tailored approaches to treat various senescence‐associated conditions. We acknowledge, however, that our validation of the *p21*‐*ATTAC* model was done in a specific condition, radiation‐induced osteoporosis, and additional studies evaluating this model in other tissues and conditions associated with senescence need to be done.

Our findings are consistent with a previous report that found that in the absence of *p21^Cip1^*, mouse embryos were impaired in their ability to undergo senescence following radiation (Brugarolas et al., 1995). In addition, the time course of induction of *p21^Cip1^* or *p16^Ink4a^* does differ following radiation both *in vitro* (López‐Domínguez et al., 2021) and *in vivo* (Chandra et al., 2020). Thus, in cultured mouse dermal fibroblasts, levels of *p21^Cip1^* mRNA were induced within 3 h of radiation, whereas *p16^Ink4a^* transcripts increased much later, ~50‐h post‐radiation (López‐Domínguez et al., 2021). In line with these findings, we recently reported that *in vivo* in mouse bones, *p21^Cip1^* expression increased within 1 day following radiation, whereas *p16^Ink4a^* expression increased gradually over the course of several weeks (Chandra et al., 2020). Thus, the earlier induction of *p21^Cip1^* following radiation both *in vitro* and *in vivo* would be consistent with the p21^Cip1^ pathway being a primary driver of cellular senescence following radiation.

Because both the *p21*‐*ATTAC* and the *p16*‐*INK*‐*ATTAC* transgenes express *Egfp*, we could monitor the induction of each transgene following radiation and the clearance of cells expressing each transgene by AP20187. As shown in Figure 1c, we could not only visualize the expression of the *Egfp* transgene, but also colocalize it with *p21^Cip1^* expression in the *p21*‐*ATTAC* model. Interestingly, the *Egfp* foci per cell showed parallel changes with radiation and AP20187 treatment to those observed for the *Egfp* transgene using qRT‐PCR (Figure 1b). These data demonstrated that although radiation induced both transgenes in bone and AP20187 cleared *p21^Cip1^*‐ and *p16^Ink4a^*‐expressing cells (which also expressed *Egfp*), senescent osteocytes, assessed using the TAF assay (Chandra et al., 2020; Farr et al., 2016), were only reduced in the *p21*‐*ATTAC* mice. These data further demonstrate that although both our transgene promoters and the endogenous *p21^Cip1^* and *p16^Ink4a^* promoters are upregulated following radiation (Chandra et al., 2020; López‐Domínguez et al., 2021), it is *p21^Cip1^* that drives senescence in radiation‐induced bone damage. Clearance of these cells prevents osteocyte senescence and the downstream consequences (i.e., increased SASP and bone loss).

We also assessed whether the selective clearance of *p21^Cip1^*‐ or *p16^Ink4a^*‐expressing cells affected SASP expression. Interestingly, while radiation‐induced upregulation of *Il6*, *Mmp12*, *Ccl2*, and *Ccl7* was prevented by clearance of *p21^Cip1^*‐expressing cells, the clearance of *p16^Ink4a^*‐expressing cells did not affect these SASP markers. Conversely, the radiation‐induced upregulation of *Ccl4* (macrophage inflammatory protein‐1β (MIP‐1β)) was attenuated following clearance of *p16^Ink4a^*‐, but not *p21^Cip1^*‐expressing cells. These data further support that p21^Cip1^ and p16^Ink4a^ are non‐redundant pathways in our radiation‐induced osteoporosis model.

We observed three unique populations of bone cells post‐radiation, one expressing *p21^Cip1^*, one expressing *p16^Ink4a^*, and the third, much smaller population expressing both *p21^Cip1^* and *p16^Ink4a^*. Interestingly, clearance of *p21^Cip1^*‐expressing cells in *p21*‐*ATTAC* mice following radiation and activation of the transgene by AP20187 reduced *p21^Cip1^*‐expressing cells, as expected, but failed to reduce *p16^Ink4a^*‐ or *p16^Ink4a^*/*p21^Cip1^* dual‐expressing cells, although the caveat here is that due to the small number of *p16^Ink4a^*/*p21^Cip1^* dual‐expressing cells, our ability to detect changes in this population was likely limited. Nonetheless, these data do indicate that *p21^Cip1^* and *p16^Ink4a^* have independent roles in radiated bones and, based on the data noted above, the time course of increases in *p21^Cip1^* versus *p16^Ink4a^* may also be of crucial importance.

The *in vivo* genetic clearance of *p16^Ink4a^*‐expressing senescent cells in *INK*‐*ATTAC* mice has been used as an important tool to study primary mechanism(s) associated with several age‐related conditions, including osteoarthritis and osteoporosis (Baker et al., 2016, 2011; Farr et al., 2017; Jeon et al., 2017; Palmer et al., 2019; Xu et al., 2015, 2018). Abrogation of CDKi's does come with the concern over lowering the threshold for neoplastic transformation; however, senescent cell deletion of *p21^Cip1^* in various mouse models of premature aging did not increase tumor incidence (Benson et al., 2009). Moreover, in the case of radiation‐induced osteoporosis, approaches to pharmacologically clear senescent cells and perhaps more specifically, *p21^Cip1^*‐expressing senescent cells, are likely to involve short‐term treatment, minimizing concerns regarding tumorigenesis.

Bone marrow adiposity is negatively associated with bone mass across species (Devlin & Rosen, 2015). Using lineage tracing of mesenchymal cells, we previously reported that the decline in BMSCs following radiation was inversely correlated with increases in bone marrow adipocytes (Chandra et al., 2017). In a recent study, we demonstrated that radiation‐induced senescent cells appear within the first‐day post‐radiation, while bone marrow adipocytes appear later (Chandra et al., 2022). Interestingly, AP20187‐induced clearance of *p21^Cip1^*‐expressing cells in the *p21*‐*ATTAC* mice significantly reduced marrow adiposity in radiated bones compared with the vehicle‐treated, radiated bones. Conversely, and consistent with the skeletal changes in the two models, clearance of *p16^Ink4a^*‐expressing senescent cells, which has been shown to negatively regulate marrow adiposity in an aging (chronic senescence) *p16*‐*INK*‐*ATTAC* model (Farr et al., 2017), did not have any effect on radiation‐induced marrow adiposity.

Our group has previously demonstrated that clearance of *p16^Ink4a^*‐expressing senescent cells using the *p16*‐*INK*‐*ATTAC* model prevents age‐related bone loss in mice (Farr et al., 2017). Whether clearance of *p21^Cip1^*‐expressing senescent cells using the *p21*‐*ATTAC* mice will also prevent age‐related bone loss remains to be experimentally defined, but our data would indicate that, in contrast to aging, clearance of *p16^Ink4a^*‐expressing senescent cells does not prevent bone loss following radiation, which appears to be driven principally by *p21^Cip1^*‐expressing cells. Clearly, further studies using both the *p16*‐*INK*‐*ATTAC* and *p21*‐*ATTAC* mice with aging and other conditions are needed to address the specific roles of *p16^Ink4a^*‐*vesrus p21^Cip1^*‐expressing cells in mediating senescence in these conditions.

In summary, our studies dissect p21^Cip1^‐ versus p16^Ink4a^‐mediated senescence in radiation‐induced osteoporosis, an established senescence‐associated condition (Chandra et al., 2020). Using novel genetic models, our studies provide the first unequivocal demonstration of a dissociation *in vivo* between these pathways in driving cellular senescence. These studies also advance our understanding of the underlying mechanism(s) of radiation‐induced bone damage, demonstrating that *p21^Cip1^*‐expressing senescent cells and their secretome regulate the early changes following radiation exposure to bone. The data presented here represent a step toward identifying senolytics that may be specific to different inducers of senescence‐mediated bone loss or other morbidities. Taken together, our studies identify a novel mechanism for radiation‐induced bone damage and validate a new mouse model that can be used to study senescence‐related tissue dysfunction in aging and disease conditions where p21^CIp1^ may play a dominant role.

## 4 MATERIALS AND METHODS

### 4.1 Animal studies

Animal studies were approved by the Institutional Animal Care and Use Committee at Mayo Clinic. All animals were housed in our facility at 23 to 25°C with a 12‐h light/dark cycle and were fed with standard laboratory chow (PicoLab® Rodent Diet 20 #5053; LabDiet, St. Louis, MO, USA) with free access to water.

The generation and characterization of the *p16*‐*INK*‐*ATTAC* transgenic mouse line (devised by T. Tchkonia, J. van Deursen, D. Baker, and J.L. Kirkland) have been described previously (Baker et al., 2011). The *p21*‐*ATTAC*‐attB transgenic plasmid was constructed as follows (Figure S1b). The pBT378 vector (provided by Tasic et al., 2011) was digested with PmeI and SwaI, to remove all DNA sequences between the two attB recombination sites, and a Geneblock (Integrated DNA Technologies, Coralville, IA) containing a single MluI site was inserted using the Gibson Assembly Master Mix (New England Biolabs, Ipswitch, MA). Next, a 3.8‐kb MluI/BssHII fragment, containing FKBP–Casp8 and IRES‐EGFP sequences from the *p16*‐*INK*‐*ATTAC* transgenic construct (devised by P. Scherer) (Pajvani et al., 2005), was cloned into the MluI site. Finally, a 3.2‐kb genomic fragment, containing promoter sequences directly upstream of mouse *Cdkn1a* variant 1 (NM_007669), was PCR amplified from mouse genomic DNA and cloned into the PmeI site using Gibson Assembly, to produce the final *p21*‐*ATTAC*‐attB transgenic plasmid. Site‐specific integrase‐mediated transgenesis (Tasic et al., 2011) (Figure S1b ) was performed at the Transgenic Mouse Facility (Stanford, CA) to insert the attB‐flanked sequences from the transgenic construct into the *Rosa26* locus on mouse chromosome 6, to produce the final *p21*‐*ATTAC* mouse model.

The mice received focal radiation as described previously (Chandra et al., 2020). Briefly, mice received a dose of 24 Gy (6.6 Gy/min) on day 0, delivered focally to 5mm of the femoral metaphyseal region using X‐Rad‐SmART (Precision X‐Ray Inc. [PXi], North Branford, CT, USA). The left femur was outside the radiation area and served as control. Starting from day 1 following radiation, the *p21*‐*ATTAC* and the *p16*‐*INK*‐*ATTAC* mice received twice weekly doses of vehicle or AP20187 (10 mg/kg) delivered intraperitoneally, respectively. The animals were injected with two fluorescent labels, Alizarin and Calcein, 9 and 2 days before tissue harvest. The animals were euthanized on day 42 for the various assessments.

### 4.2 Methyl methacrylate tissue embedding and histology

Radiated and non‐radiated femurs were processed as described previously (Chandra et al., 2020). Briefly, at day 42 post‐radiation the bones were processed for routine methyl methacrylate (MMA) embedding. Non‐decalcified femurs were sectioned into 5 μm sections that were used for static histomorphometry and TIF assay, while 8 μm sections were used for dynamic histomorphometry.

### 4.3 Micro‐computed tomography (μCT) analysis

Bones were harvested 42‐day post‐focal radiation and scanned using μCT (vivaCT 40, Scanco Medical AG, Brüttisellen, Switzerland). The distal femur was scanned corresponding to a 1–5 mm area above the growth plate. All images were first smoothed by a Gaussian filter (sigma = 1.2, support = 2.0) and then thresholded corresponding to 30% of the maximum available grayscale values. Volumetric bone mineral density (vBMD), bone volume fraction (BV/TV), trabecular thickness (Tb.Th), trabecular separation (Tb.Sp), trabecular number (Tb.N), and structure‐model index (SMI) were calculated using 3D standard microstructural analysis.

### 4.4 Quantitative RT‐PCR

Bones were collected for mRNA isolation and qRT‐PCR as described previously (Chandra et al., 2020). Briefly, a 5‐mm region below the growth plate of the distal metaphyseal femur was cut out from the R and NR legs. After removal of muscle tissue, the bone samples were homogenized and total RNA was isolated using RNeasy Mini Columns (QIAGEN, Valencia, CA). cDNA was generated from mRNA using the High‐Capacity cDNA Reverse Transcription Kit (Applied Biosystems by Life Technologies, Foster City, CA) according to the manufacturer's instructions, and RT‐qPCR was performed as described in our previous studies(Chandra et al., 2020). All primer sequences have been authenticated in previous studies (Chandra et al., 2020). Primers were designed so that they overlapped two exons. A detailed list of primer sequences is provided in Table S2.

### 4.5 Static and dynamic histomorphometry

Static histomorphometry was performed using 5 μm Goldner's trichrome‐stained sections, counting osteoblast numbers (N.Ob) and osteoclast numbers (Oc.N)/mm of bone surface. Cuboidal shaped cells located on bone surfaces were characterized as osteoblasts, while multinucleated cells on the bone surface with a well‐defined pit were characterized as osteoclasts. The region of interest for all histomorphometric assessments was within the 5mm region of radiation and a corresponding 5mm region in the contralateral leg, below the growth plate. An area of 1 mm^2^ 0.6 mm below the growth plate was used for static and dynamic histomorphometric analysis. Adipocyte numbers (Ad.N) and volume (Ad.V) were quantified in an area of 1.72 mm^2^, 0.6 mm below the growth plate, normalized against bone marrow area (total tissue area minus bone area). Dynamic histomorphometry was performed on unstained deplasticized sections, and mineral apposition rate (MAR) was calculated as described previously (Chandra et al., 2020). Briefly, mineralized bone was identified by alizarin (red/orange) and calcein (green) fluorescent dyes, with mineralization being a marker of functional osteoblasts, and the distance between the two dye fronts indicative of new bone formed in 7 days, calculated by MAR. Both static and dynamic histomorphometry was performed using the OsteoMeasure™ Histomorphometry System (OsteoMetrics™, Inc.) with an Olympus Dotslide motorized microscope system.

### 4.6 Telomere fluorescence in situ hybridization (FISH) and DNA damage

Paraffin‐embedded bone sections were de‐paraffinized followed by a serial ethanol series in 100%, 90% and 70% ethanol, with final hydration in PBS. Antigen retrieval was done in 0.01 M citrate buffer (pH 6.0) at 95°C for 15 min, and cooled slides were washed in water and PBS for 5 min each. Subsequently, a blocking step was performed (normal goat serum 1:60 in PBS/BSA, #S‐1000; Vector Laboratories) for 30 min at room temperature (RT) followed by incubation with a primary antibody (γ‐H2A.X, Cell Signaling, mAb #9718) overnight at 4°C. Slides were washed three times with PBS for 5 min and incubated for 60 min with a species‐specific secondary antibody (no. PK‐6101; Vector Lab). Sections were then washed 3 times in PBS for 5 min, and Cy5 Streptavidin (1:500 in PBS, No: SA‐1500, Vector Lab) was applied for 25 min followed by three washes in PBS, followed by cross‐linking with 4% paraformaldehyde for 20 min and dehydration in graded icy‐ethanol. Sections were denatured for 10 min at 80°C in hybridization buffer [70% formamide (Sigma UK), 25 mM MgCl2, 0.1 M Tris (pH 7.2), 5% blocking reagent (Roche, Germany)] containing 2.5 μg/ml Cy‐3‐labeled telomere‐specific (CCCTAA) peptide nucleic acid probe (Panagene), followed by hybridization for 2 h at RT (minimum) in a humidified chamber in the dark. Next, slides were washed once with 70% formamide in 2 × SSC for 10 min, followed by 1 wash in SSC buffer and PBS for 10 min. Sections were mounted with DAPI (ProLong™ Diamond Antifade Mountant, Invitrogen, P36962). In‐depth Z‐stacking was used (a minimum of 135 optical slices using a 63× objective). Number of TAFs/cell was assessed by manual quantification of partially or fully overlapping (in the same optical slice, Z) signals from the telomere probe and γH2A.X in z‐by‐z analysis. Images were deconvolved with blind deconvolution in AutoQuant X3 (Media Cybernetics).

### 4.7 RNA in situ hybridization (RNA‐ISH)

RNA‐ISH was performed *per* the RNAScope protocol from Advanced Cell Diagnostics Inc. (ACD): RNAScope Multiplex Fluorescent Assay v2. Briefly, the assay allows simultaneous visualization of up to 4 RNA targets, with each probe assigned to a different channel (C1, C2, or C3 or C4). p21 (CDKN1A) signal amplified using the HRP‐C1 linked with a secondary fluorophore, Opal 570 (detected in the Cy3 range), followed by a subsequent detection of p16/p19 (CDKN2A) –c3 or EGFP‐c2 probe, the signal is amplified by the HRP‐C3 or HRP‐C2 (respectively) linked with a secondary fluorophore, Opal 650 (detected in the Cy5 range). Tissue sections were then mounted using ProLong Gold Antifade Mountant with DAPI (Invitrogen). Sections were imaged using in‐depth Z‐stacking. RNA‐ISH‐positive cells on the bone surface were quantified normalized against total cells on the bone surface.

### 4.8 RNA in situ hybridization (RNA‐ISH) and immunofluorescence

Briefly after the RNAish last amplification and blocking for 15 min with the HRP blocker (following manufacturer instructions), slides were washed for three times in PBS (5 min each), then blocked in normal goat serum (1:60) in BSA/PBS for 30 min, and incubated with primary antibodies 1:200 overnight at 4°C with either: CD11b (MAS‐17857) or CD3e (14‐0032‐82) or Cd19 (0194‐82) (rat, Thermo Fisher). Following three PBS washes for 5 min, sections were then incubated with a rat fluorescent secondary antibody for 1 h (goat anti‐rat 647 Alexa Fluor A‐21247, followed by PBS washes and mounted using ProLong Gold Antifade Mountant with DAPI (Invitrogen). Sections were imaged using a Leica microscope (dmi8, ×40 dry objective).

### 4.9 Statistical analysis

Sample sizes were based on previously published data (Chandra et al., 2020; Farr et al., 2017), in which statistically significant differences were observed on bone with various interventions in our laboratory. For all experiments, data are expressed as median with interquartile range. Statistical comparisons were done using two‐tailed unpaired *t* tests. For multiple comparisons in Figures 2c,d, 3b, 4b,d, and 3a,b, the data were analyzed using a two‐way ANOVA with a Tukey post hoc analysis. All statistical analyses were performed by GraphPad Prism.

## CONFLICT OF INTEREST

Patents on *p16*‐*INK*‐*ATTAC* mice and their uses are held by Mayo Clinic. This research has been reviewed by the Mayo Clinic Conflict of Interest Review Board and was conducted in compliance with Mayo Clinic Conflict of Interest policies.

## AUTHOR CONTRIBUTIONS

Abhishek Chandra, Robert J. Pignolo, and Sundeep Khosla designed the study and Abhishek Chandra, Anthony B. Lagnado, and Joshua N. Farr performed the experiments, while Abhishek Chandra, David G. Monroe, Joshua N. Farr, João F. Passos, Robert J. Pignolo, and Sundeep Khosla analyzed and interpreted the data. Abhishek Chandra, Robert J. Pignolo, and Sundeep Khosla wrote the initial manuscript, and all authors contributed ideas and revised and approved the final version of the manuscript.

## Supporting information

Supplementary Material

## Data Availability

All data will be made available on request.
