# Supplementary material for: Targeted clearance of p21‐ but not p16‐positive senescent cells prevents radiation‐induced osteoporosis and increased marrow adiposity
Source: Aging Cell. 2022 Apr 1;21(5):e13602. doi: 10.1111/acel.13602 (PMC9124310; doi:10.1111/acel.13602)
Supplement: Supplementary file 1 — Supplementary Material [file ACEL-21-e13602-s001.pdf]

Supplementary Figure 1

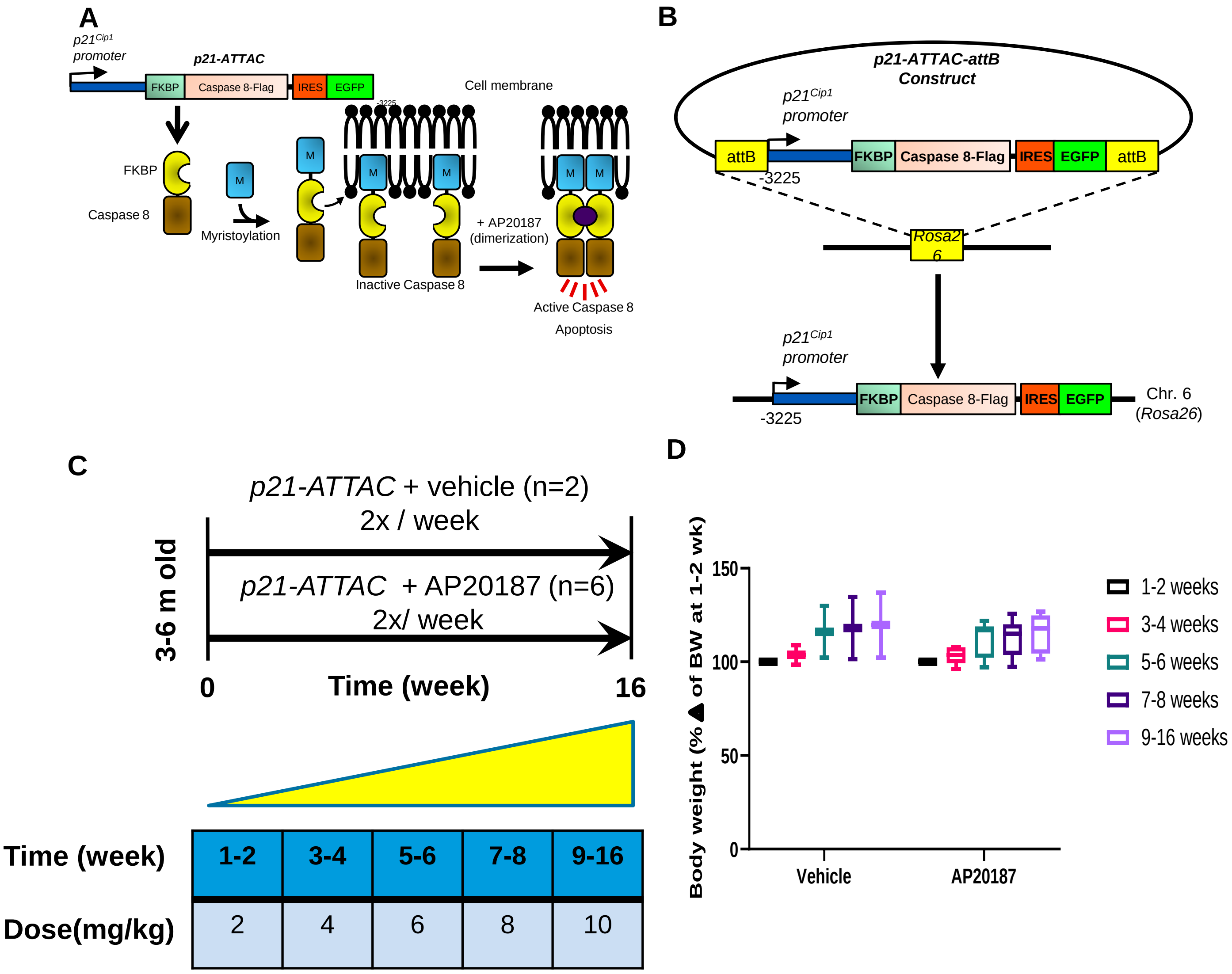

Supplementary Figure 1. Construction of the *p21-ATTAC* mouse model. (A) A schematic of the *p21-ATTAC* construct, driven by the *p21*^{Cip1}-promoter. Treatment with AP20187 initiates the dimerization of the FKBP-Caspase 8 casette and induces cell death in the *p21*^{Cip1}-expressing senescent cells; (B) Site-specific integration of the *p21-ATTAC* transgene into the *Rosa26* locus; (C) Experimental design to test AP20187 toxicity in young mice. The animals were assigned to vehicle- or AP20187-treated groups. The AP20187 was given in an increasing dose regimen, ranging from 2-10 mg/kg; (D) Body weights of vehicle- and AP20187-treated animals as percentage change from the time of the first dose. Both vehicle- and AP20187-treated animals had no physical signs of distress, frailty or other abnormalities, and both groups gained normal levels of body weight.

©2020 MFMER | slide-1

Supplementary Figure 2

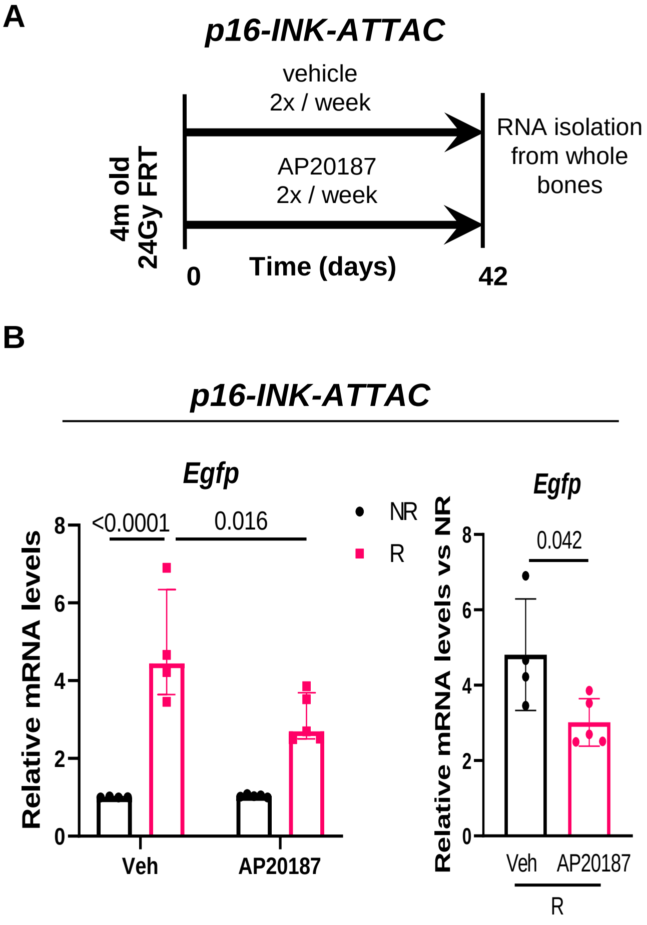

Supplementary Figure 2. Validation of *p16-INK-ATTAC* mouse models using radiation as an inducer of senescence. Schematic showing the experimental design for the *p16-INK-ATTAC* (A) mice. The right legs of the mice were radiated (24 Gy) near the femoral metaphysis (5 mm above the growth plate), while the left leg served as control. Starting from day 1 post radiation, *p21-ATTAC* and *p16-INK-ATTAC* animals received vehicle or AP20187 for 2 times week for 6 weeks. Radiated (R) and non-radiated (NR) femurs at 42 days post-radiation were collected for qRT-PCR. (B) The *Egfp* transgene was activated in R bones, and the expression levels were significantly reduced in the AP20187-treated animals. Statistical comparisons between the groups (left panel in B) was done by an ordinary two-way ANOVA, with a Tukey's post-hoc analysis. (Right panel in B) The *Egfp* expression was normalized to the NR control leg for each animal. Statistical comparison was done using a two-tailed unpaired t-test between the Veh-R and AP20187-R bones.

Supplementary Figure 3

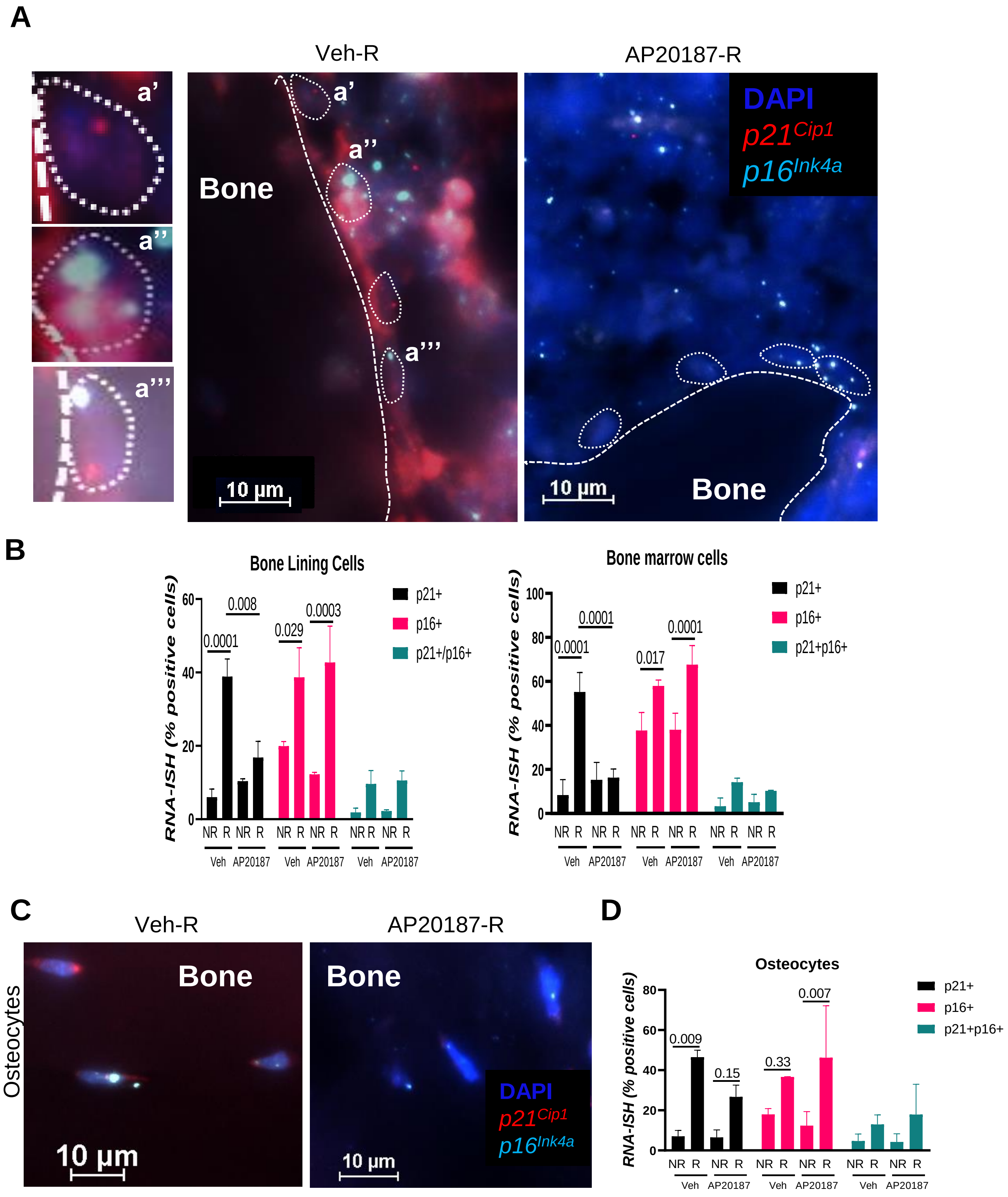

Supplementary Figure 3. Validation of the *p21-ATTAC* mouse model by RNA *in situ* hybridization. (A) The right legs of *p21-ATTAC* mice were radiated (24 Gy) near the femoral metaphysis (5 mm above the growth plate), while the left leg served as control. The animals were assigned to vehicle- or AP20187-treated groups. R- and NR-femurs at 42 days post-radiation were collected for histology (fixed, decalcified, and processed for paraffin embedding). RNA-*in situ* hybridization (RNA-ISH) was performed using probes against $p21^{Cip1}$ (shown in red) and $p16^{Ink4a}$ (shown in light blue) transcripts. Cells on the bone surface were defined as bone lining cells (BLC, dashed outlined cells) and three populations of cells, one expressing $p21^{Cip1}$ (a'), a second expressing $p16^{Ink4a}$ (a'', b' and b''), and a third expressing both $p21^{Cip1}$ and $p16^{Ink4a}$ (a'''). The three populations were quantified in the bone lining cells and in the bone marrow cells (shown in B). (C) Similar estimates of p21- and p16- expressing osteocytes were made, and their representation is shown in (D). Statistical comparisons were calculated using an ordinary two-way ANOVA, with a Tukey's post-hoc analysis.

Supplementary Figure 4

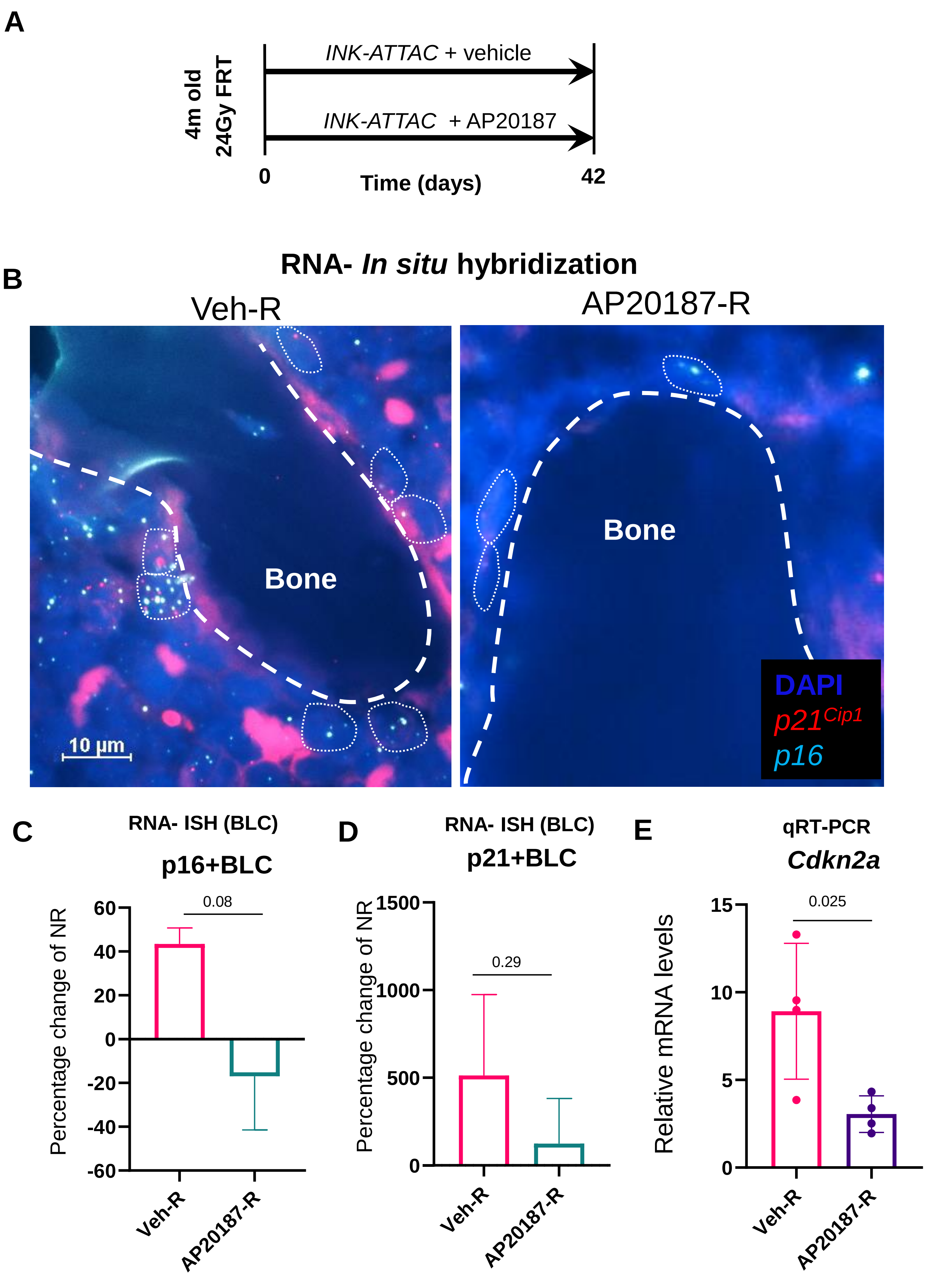

Supplementary Figure 4. Validation of the *p16-INK-ATTAC* mouse model by RNA *in situ* hybridization. (A) The right legs of *p16-INK-ATTAC* mice were radiated (24 Gy) near the femoral metaphysis (5 mm above the growth plate), while the left leg served as control. The animals were assigned to vehicle- or AP20187-treated groups. R- and NR-femurs at 42 days post-radiation were collected for histology (fixed, decalcified, and processed for paraffin embedding). (B) RNA-*in situ* hybridization (RNA-ISH) was performed using probes against *p21*^{Cip1} (shown in red) and *p16*^{Ink4a} (shown in light blue) transcripts. Cells on the bone surface were defined as bone lining cells (BLC, dashed outlined cells) and two populations of cells were quantified, one expressing *p16*^{Ink4a} (C) and a second expressing *p21*^{Cip1} (D). (E) Using qRT-PCR, *Cdkn2a gene* expression levels were detected in R-femurs from vehicle- or AP20187-treated mice. Statistical comparisons were calculated using a two-tailed t-test.

Supplementary Figure 5

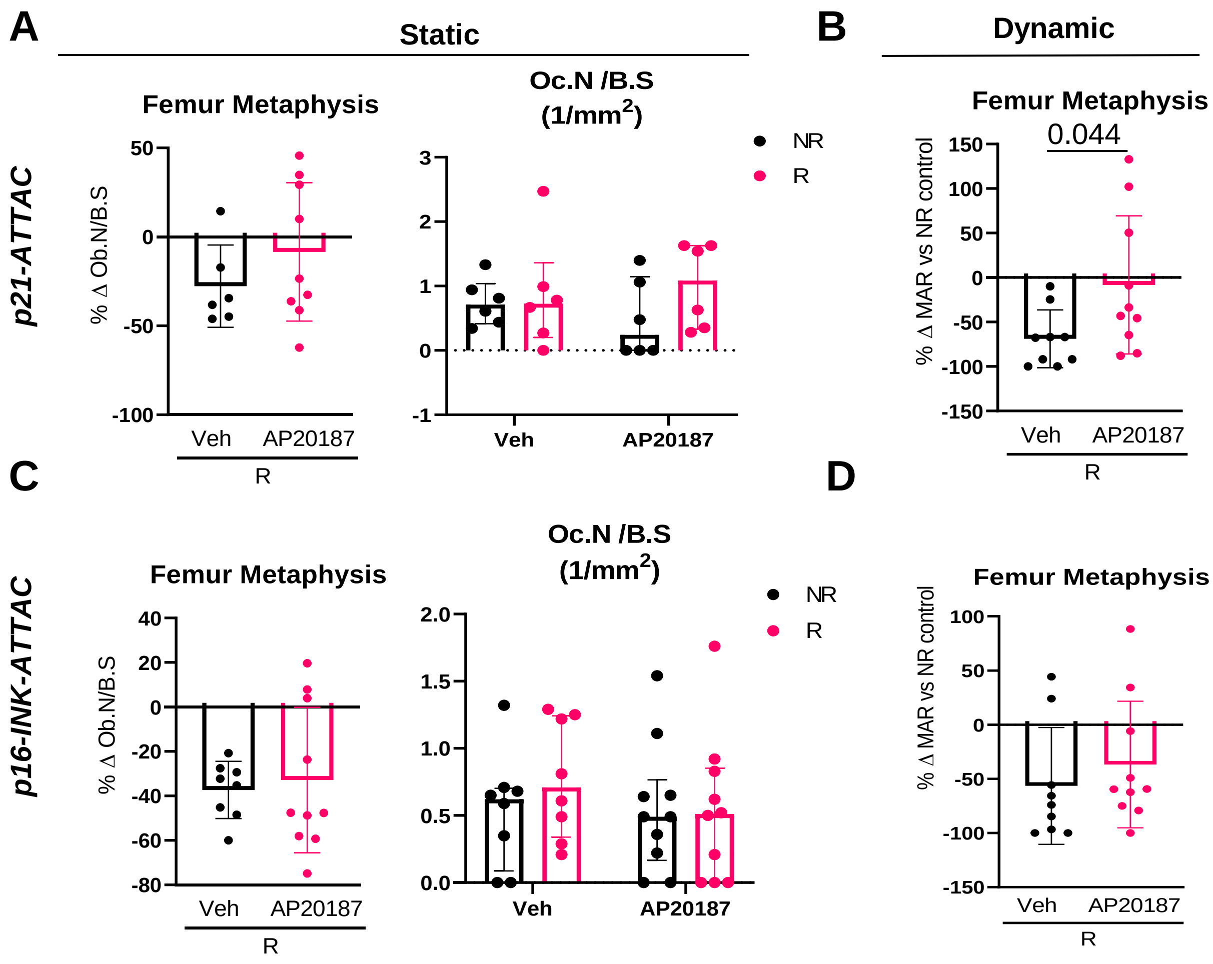

Supplementary Figure 5. Assessment of bone formation in radiated bones following clearance of *p21*^{Cip1}- and *p16*^{Ink4a}-expressing cells. The right legs of *p21-ATTAC* and *p16-INK-ATTAC* mice were radiated (24 Gy) near the femoral metaphysis (5 mm above the growth plate), while the left legs served as control. The animals received either vehicle or AP20187 as described above. The samples previously scanned for μCT were processed for MMA embedding. (A, C) Osteoblast and osteoclast numbers were quantified in 5 μm Goldner's trichrome stained sections, and mineral apposition rate (MAR) (μm/day) was calculated using alizarin and calcein stained 8 μm MMA embedded sections. (B, D) Percentage change in radiated bones was normalized against control and comparisons in MAR were done between vehicle- and AP20187-treated bones. Statistical comparisons were done using a two-tailed unpaired t-test between the Veh-R and AP20187-R bones. For OC.N/B.S (A, C) statistical comparisons were done using an ordinary two-way ANOVA, with a Tukey's post-hoc analysis.

Supplementary Figure 6

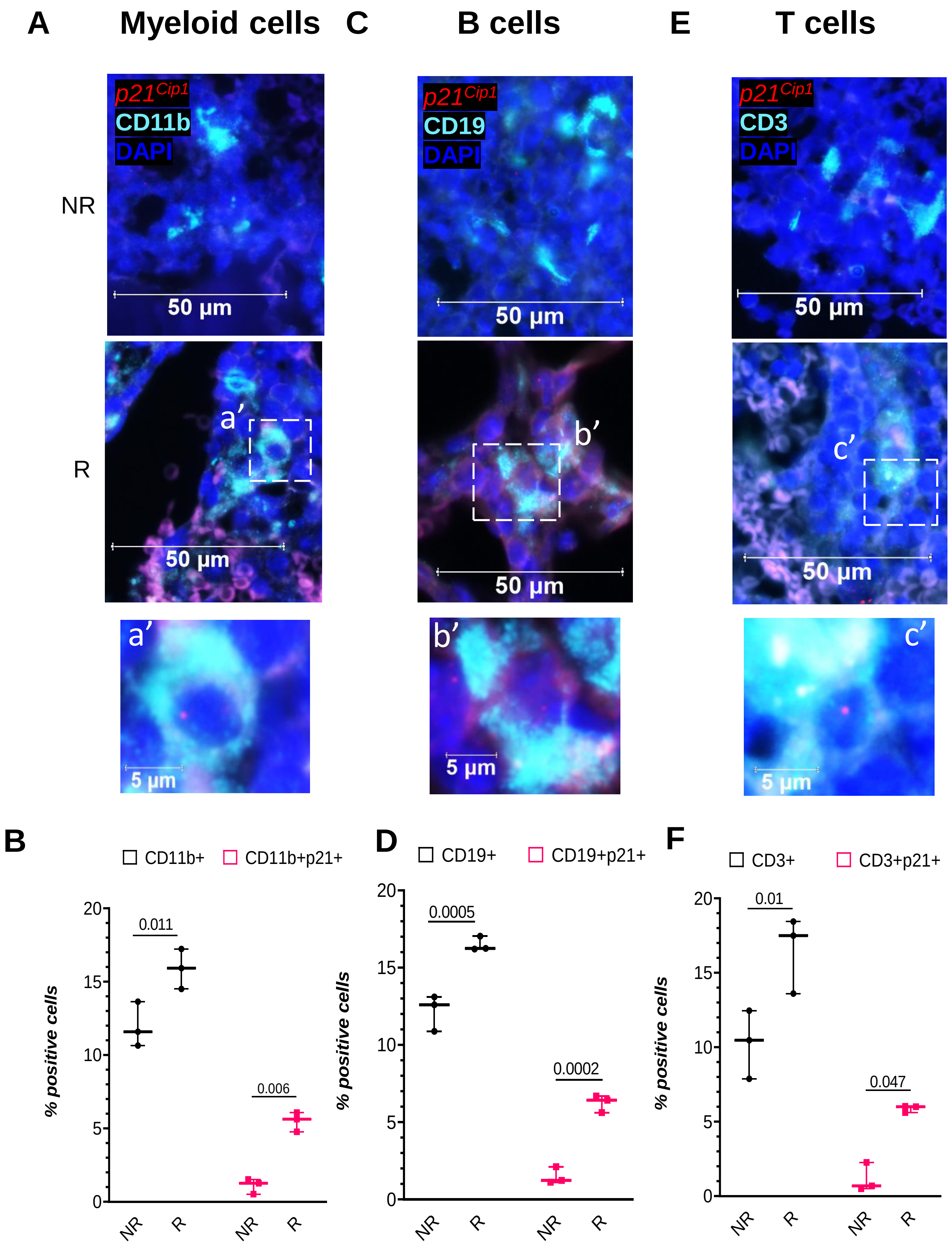

Supplementary Figure 6. Identifying *p21* expressing bone marrow cells in radiated bone. The right legs of C57Bl/6 mice were radiated (24 Gy) near the femoral metaphysis (5 mm above the growth plate), while the left leg served as control. R- and NR-femurs were collected at 7 days post-radiation for histology (fixed, decalcified, and processed for paraffin embedding). To identify *p21*^{Cip1} expressing bone marrow cells we performed RNA-*in situ* hybridization (RNA-ISH) using probe against *p21*^{Cip1} (shown in red) and antibodies against cell surface markers to identify CD11b+ myeloid cells (A), CD19+ B cells (C) and CD3+ T cells (E), and the percentage of these cells were quantified (B, D and F). Statistical comparisons were calculated using an ordinary two-way ANOVA, with a Tukey’s post-hoc analysis.

Supplementary Table S1. List of SASP factors evaluated

Gene Symbol	Description
Ccl1	C-C motif chemokine ligand 1
Ccl2	C-C motif chemokine ligand 2
Ccl4	C-C Motif Chemokine Ligand 4
Ccl5	C-C Motif Chemokine Ligand 5
Ccl7	C-C Motif Chemokine Ligand 7
Cxcl1	C-X-C Motif Chemokine Ligand 1
Cxcl2	C-X-C Motif Chemokine Ligand 2
Cxcl9	C-X-C Motif Chemokine Ligand 9
Cxcl13	C-X-C Motif Chemokine Ligand 13
Cxcl16	C-X-C Motif Chemokine Ligand 16
Il6	Interleukin 6
Il10	Interleukin 10
Il18	Interleukin 18
Icam1	Intercellular Adhesion Molecule 1
Igfbp4	Insulin Like Growth Factor Binding Protein 4
Mmp12	Matrix Metallopeptidase 12

Supplementary Table S2. Mouse primer sequences

	**Forward Primer sequence (5'-3')**	**Reverse Primer Sequence (5'-3')**
Actb	AATCGTGCGTGACATCAAAGAG	GCCATCTCCTGCTCGAAGTC
Egfp	ACAGCCACAACGTCTATATC	CTTGAAGTTCACCTTGATGC
Il6	ACCACGGCCTTCCCTACTTC	TTGGGAGTGGTATCCTCTGTGA
Mmp12	GTGCCCGATGTACAGCATCTT	GGTACCGCTTCATCCATCTTG
Ccl4	AGGGTTCTCAGCACCAATGG	CCGGGAGGTGTAAGAGAAACAG
Ccl5	GCCCACGTCAAGGAGTATTTCT	ACAAACACGACTGCAAGATTGG
Ccl7	CCCTGGGAAGCTGTTATCTTCA	CTGATGGGCTTCAGCACAGA
Tuba1	GGTTCCCAAAGATGTCAATGCT	CAAACTGGATGGTACGCTTGGT
